# Is the colposcopic lesion size a predictor of high-grade lesions in young patients?

**DOI:** 10.31744/einstein_journal/2024AO0462

**Published:** 2024-06-21

**Authors:** Beatriz Mokwa dos Santos, Edward Araujo, Rita Maira Zanine

**Affiliations:** 1 Universidade Federal do Paraná Hospital das Clínicas Department of Obstetrics and Gynecology Curitiba PR Brazil Department of Obstetrics and Gynecology, Hospital das Clínicas, Universidade Federal do Paraná, Curitiba, PR, Brazil.; 2 Universidade Federal de São Paulo Escola Paulista de Medicina Department of Obstetrics São Paulo SP Brazil Department of Obstetrics, Escola Paulista de Medicina, Universidade Federal de São Paulo, São Paulo, SP, Brazil.

**Keywords:** Colposcopy, Squamous intraepithelial lesions of the cervix, Size perception, Injury Severity Score, Age factor

## Abstract

**Objective:**

This study aimed to evaluate whether severity changes with colposcopic lesion size, regardless of age.

**Methods:**

This retrospective comparative study reviewed the records of 428 women with altered cytopathology reports who were directed by primary health care. Only those women with colposcopic alterations were evaluated (n=411). Histopathological analyses were restricted to patients who underwent excisional treatment (n=345). According to their age, they were grouped into the following: <21, 21–24, 25–35, and >35 years, and also, ≤24 and ≥25 years. The cytopathological, colposcopic, and histopathological findings were grouped according to severity. Lesion size was subjectively assessed from the colposcopic drawing recorded in the chart and according to the number of quadrants of the total cervical surface affected by colposcopic alterations in the transformation zone. Statistical significance was set at p<0.05.

**Results:**

The evaluations suggested that the lesion size was directly related to the severity of the cytopathology, colposcopy, and histopathology reports for the age groups ≤24 or ≥25 years. We observed associations between lesion size and severity of the cytopathology (≤24 years, p=0.037) and histopathology (≥25 years, p=0.003) findings.

**Conclusion:**

The size of the lesion was directly related to the severity of the histopathological lesion in patients aged ≥25 years and cytopathological in patients aged ≤24 years.

## In Brief

Santos et al. demonstrated that the size of the lesion was directly related to the severity of the cytopathological, colposcopy, and histopathological reports for the age groups ≤24 or ≥25 years. They observed a greater association between lesion size and cytopathology severity in patients ≤24 years of age (p=0.037) and with histopathology severity in women ≥25 years of age (p=0.003).

## Highlights

▪ The size of colposcopic lesions appears to be directly related to lesion severity.

▪ The size of the lesion is directly related to the severity of the histopathological findings in patients ≥25 years old.

▪ The size of the lesion is directly related to the severity of the cytopathological findings in patients aged ≤24 years.

## Graphical abstract

**Figure:**
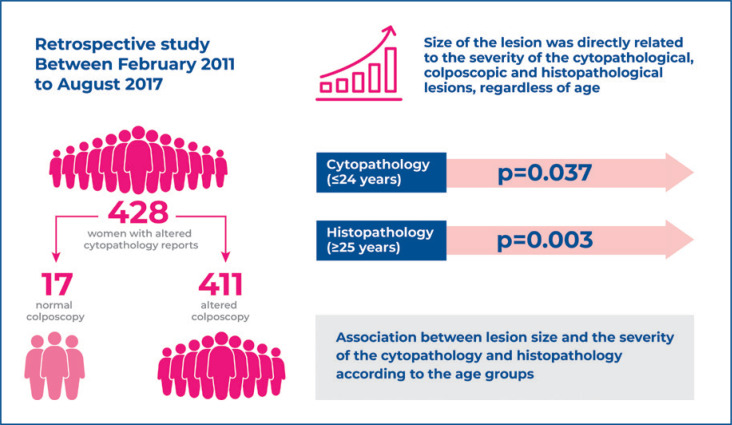

## INTRODUCTION

Cervical cancer is a major public health concern in Brazil. In 2023, 17,010 new cases are estimated, representing an adjusted incidence rate of 13.25 cases per 100,000 women.^(1)^

The high incidence and mortality, principally in less-developed regions, reveal inequalities between different socioeconomic groups and exposure to the risk factors present in these areas. Its occurrence stems from poor living conditions such as a lack of access to health services, especially population screening programs.^(2)^ Carcinoma of the cervix can be prevented by screening. Optimal prevention strategies should identify cervical abnormalities that are likely to progress to invasive cancer while avoiding destructive treatment of abnormalities not destined to become cancerous.^(3)^

Since 2011, the Brazilian Guidelines for the Screening of Cervical Cancer have advocated that screening begin at the age of 25 years.^(4)^ It should be emphasized that the benefits of screening in adolescents and young women may be limited; however, if screening is performed, minimizing unnecessary treatment damage becomes a concern.^(5)^ There is a low incidence of cancer among adolescents and young women is low, and there is evidence that screening is less efficient for detecting lesions.^(3,6,7)^ Sasieni et al.^(8)^ showed that screening in women aged 20–24 years did not affect cervical cancer incidence until the age of 30 years. This finding supports the recommendation of observation over treatment for young women (aged 13–25) with grade 2 cervical intraepithelial neoplasia (CIN), as 68% of these cases resolve on their own within 3 years.

The literature reports that screening and treatment of women below the age of 21 years continues to occur. In Brazil, cervical carcinoma screening is opportunistic, and women aged less than 24 years are still being screened.^(9)^ The Brazilian Guidelines consider special situations in which women are inadvertently included in screening programs to better guide professionals who work in secondary health centers.^(4,7)^

Inadequate sampling and sample transfer are the main causes of false negative results in conventional oncotic cytopathology.^(10)^ Furthermore, errors in the cytopathologist's interpretations, the type of spatula and cytobrush, and the collector's experience also influenced the cytological findings.^(11)^ Researchers assume that cytopathologies with false-negative results are more common in small lesions, regardless of age. Adolescents and young women are believed to have smaller lesions because of their shorter natural histories.^(12)^

The mechanisms that explain the absence of CIN in the histopathology of excisional examinations after a positive incisional biopsy remain unknown, poorly studied, and controversial. Approximately 13–30% of women with histopathologies previously confirmed by biopsy have surgical specimens without lesions (negative or normal). Although excision of the transformation zone (ETZ), the main technique for treating CIN, is considered a conservative and safe procedure, it is associated with various risks of hemorrhage, cervical stenosis, sub-infertility, and gestational morbidity. Therefore, the factors associated with the absence of disease in the histopathology of specimens need to be identified. The two main hypotheses currently being considered are either regression of the CIN between the initial evaluation and the biopsy, or complete removal of the lesion during the biopsy itself.^(13)^ However, other factors have been of clinical interest, such as lesion size (occupying up to one quadrant of the cervix),^(14,15)^ lesion characteristics, HPV genotype, and low viral load, which could be associated with greater CIN regression.^(13)^

The size of the lesion, in turn, has been neglected in studies on the natural history of CIN and cervical cancer screening. However, this also explains the discrepancies between cytology, colposcopy, and histopathology.^(11)^ Furthermore, it is presumed that small lesions may be at an earlier stage of the natural history of the disease or have a slower progression and low probability of transforming into invasive cancers.^(11,16)^ Therefore, small lesions observed during colposcopy do not require surgery.^(13)^

The lesion size is an important predictor of cytopathological and colposcopic accuracy. Cervical intraepithelial neoplasia 3 false negatives occurred most frequently in cervicovaginal smears of small lesions.^(14,17)^ Giles et al.^(17)^ suggested that insufficient exfoliation of abnormal cells capable of being detected by cytopathology may explain the 58% false negative rate in the cytopathology of small lesions.

Colposcopy accuracy is affected by the presence of small lesions. Colposcopic sensitivity decreases when lesions are small and is less sensitive for detecting CIN 3 or cancer when the lesion involves only one quadrant of the cervix.^(14,18–20)^ Strander et al.^(21)^ developed the Swede Score, a colposcopic index that includes the size of the lesion as a Reid index variable. The Swedish score had a specificity of 90% to exclude lesions greater than or equal to CIN 2 when the score was <5. They also showed that 70% of larger lesions had a minimum score of two, resulting in histopathological lesions greater than or equal to CIN 2.^(22)^

## OBJECTIVE

To evaluate whether the size of the lesion was related to the age and severity of cytopathological, colposcopic, and histopathological lesions.

## METHODS

This retrospective and comparative study included patients who had been directed from the primary healthcare network to undergo colposcopic examination at the Lower Genital Tract Pathology and Colposcopy Outpatient Clinic of the General Hospital of the *Universidade Federal do Paraná* (UFPR), Curitiba, Brazil, between February 2011 and August 2017. These patients presented with alterations in conventional cytopathological examinations. Colposcopies were performed under the supervision of the same examiner, who had over 40 years of experience in the field. This study was approved by the Research Ethics Committee of *Universidade Federal do Paraná* (CAAE: 54037716.1.0000.0096; # 1.508.463.

The analysis was conducted using data from 428 women with altered cytopathological reports. Only women with altered colposcopy findings (n=411) were considered for evaluation of the colposcopic findings. Histopathological analyses were restricted to patients who underwent excisional treatment (n=345). The charts of all adolescents and young women (14–24 years old) were analyzed. An age limit of 24 years was established considering the age at which screening for cervical cancer began in Brazil. This group was compared to a group of women of childbearing age who were randomly selected from hospital records.

Patients with incomplete medical records or clinical conditions that could bias or cause imprecise measurement of the uterine cervical lesion size, such as pregnant women, immunosuppressed women, menopausal women, previous history of surgery or cervical disease, inadequate colposcopy (cervix obscured by inflammation, bleeding, or scarring), and women with transformation zone type 3 (squamocolumnar junction not visible in the ectocervix), were excluded.

The patients included in this study were divided according to their age into the following groups: <21, 21–24, 25–35, and >35 years, and in two further groups: ≤24 and ≥25 years. The main variable evaluated was the size of uterine cervical lesions according to age. The lesion size was subjectively assessed from the colposcopic drawing recorded in the chart and according to the number of quadrants of the total cervical surface affected by colposcopic alterations in the transformation zone. The sum of the colposcopic abnormalities was considered.

The following data were collected from the selected cases through a manual review of medical records at the Hospital Archive Service: age, number of quadrants occupied by the lesion on the surface of the uterine cervix, cytopathological and colposcopic findings, and histopathology data.

Colposcopy was performed using a colposcopic device (DF Vasconcelos Ltd., Valença, Brazil). First, the vascular patterns of the cervix were evaluated under green light, then 5% acetic acid, and finally Lugol's solution (Schiller's test) was applied. The findings were interpreted and presented in the iconography medical records. Colpo-directed biopsies were performed using Gaylor tweezers in cases where larger colposcopic findings were identified. The biopsy specimens were fixed in formalin solution and sent for later analysis to the Anatomic Pathology Hospital of the UFPR. These women were subjected to the most appropriate excisional treatment if they were positive for high-grade cervical intraepithelial neoplasia. Women with type 1 or type 2 transformation zones underwent type 1 or type 2 excision of the transformation zone, respectively, under local anesthesia, whereas women with type 3 transformation zones underwent type 3 exertion of the processing zone, performed following the classic cold scalpel technique. Surgical specimens were marked at the 12 o'clock position, referred to the Pathology Service Hospital of the UFPR for analysis, identified, and immersed in the formalin solution.

Cytopathology findings were grouped into: "Low-Grade Group" (Atypical squamous cells of undetermined significance, possibly non-neoplastic; Low-grade Intraepithelial Lesion), "High-Grade Group" (Atypical squamous cells of undetermined significance when High-grade Intraepithelial Lesion cannot be excluded; Atypical glandular cells and High-grade Intraepithelial Lesion), and "Cancer Group," for statistical analysis.

The colposcopy findings were grouped into the following groups: "Normal Group, Low-grade Group/Minor findings" (papillary epithelium, thin acetowhite epithelium, fine or dotted mosaic), "High-grade Group/Major findings" (dense acetowhite epithelium, coarse mosaic, and dotted or cornified orifice), and "Cancer Group" (atypical vessels).

Histopathology findings were grouped into: "Normal Group" (negative or cervicitis), "Low-grade Group" (Cervical intraepithelial neoplasia 1), "High-Grade Group" (Cervical intraepithelial neoplasia 2, Cervical intraepithelial neoplasia 3, in situ carcinoma), and "Cancer Group" (micro-invasive, invader).

The results of the analyzed variables are described in terms of frequencies and percentages. The χ^2^ test or Fisher's exact test was used to evaluate the association between two categorical variables. Multiple comparisons were made using a Logistic Regression model and Wald test. For each of the variables analyzed, we tested the null hypothesis that the distributions over the variable classifications were equal for the four groups or (2 groups) defined by age group *versus* the alternative hypothesis that the distributions were not all equal.

Statistical significance was set at p<0.05. When possible, odds ratios (OR) were estimated using a 95% confidence interval. The data were analyzed using the Stata/SE v.14.1 software (Stata Corp., College Station, TX, USA).

## RESULTS

**Figure 1:**
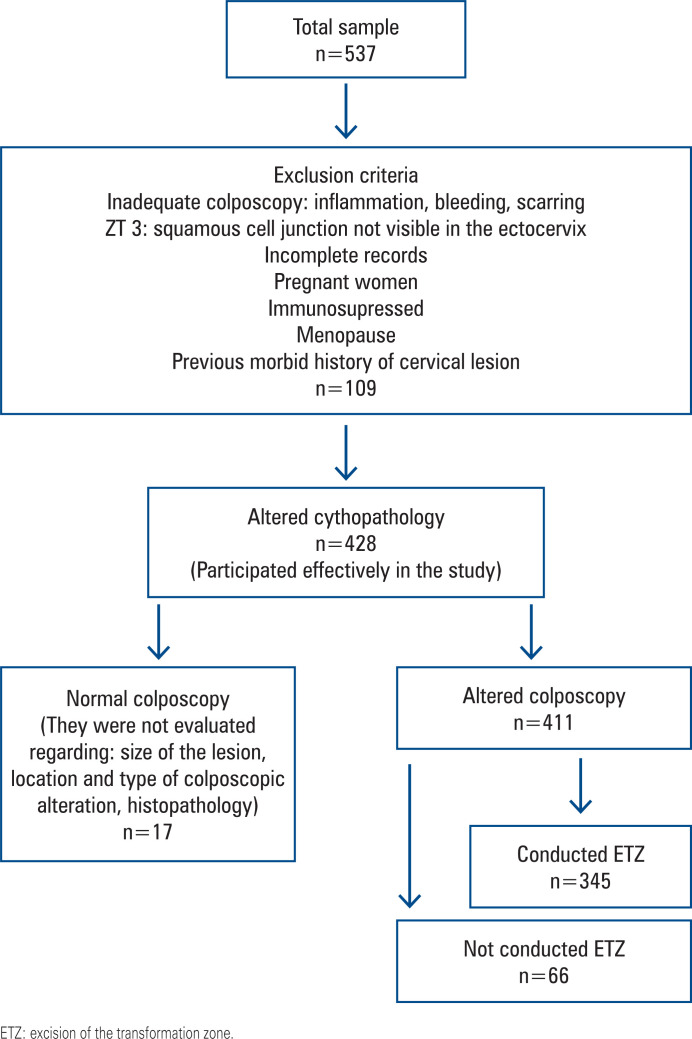
Inclusion patients selection

Figure 1 shows a flowchart of patient inclusion. The patients had a mean age of 25.8±6.9 years, with a minimum of 14 years and a maximum of 49 years.

The colposcopic size of the lesion was described as occupying one, two, three, or four quadrants of the cervix. The sample sizes, regardless of age, are presented in table 1.

**Table 1:** Sample distribution among the four age groups, the two groups, and colposcopic sizes

		n (%)
Sample distribution between the four age groups		
	<21 years	99 (23.1)
	21–24 years	140 (32.7)
	25–35 years	145 (33.9)
	>35 years	44 (10.3)
Sample distribution between the two age groups		
	≤24 years	239 (55.8)
	≥25 years	189 (44.2)
Sample distribution among the colposcopic sizes		
	1 quadrant	103 (25.1)
	2 quadrants	170 (41.4)
	3 quadrants	43 (10.5)
	4 quadrants	95 (23.1)

The evaluations suggested that the lesion was directly related to the severity of the cytopathology, colposcopy, and histopathology reports for the age groups ≤24 or ≥25 years. We observed associations between lesion size and severity of the cytopathology (≤24 years, p=0.037) and histopathology (≥25 years, p=0.003) reports (Table 2).

**Table 2:** Association between lesion size and the severity of the cytopathology, colposcopy, and histopathology reports according to the age groups

		1 quadrant	2 quadrant	3 quadrant	4 quadrant	p value*
Cytopathology (≤24 years)						0.037
	Low-grade	39 (68.4)	47 (54.7)	20 (71.4)	23 (45.1)	
	High-grade	18 (31.6)	39 (45.3)	8 (28.6)	28 (54.9)	
	Total	57	86	28	51	
Cytopathology (≥25 years)						0.567
	Low-grade	21 (45.7)	41 (48.8)	5 (33.3)	17 (38.6)	
	High-grade	25 (54.3)	43 (51.2)	10 (66.7)	27 (61.4)	
	Total	46	84	15	44	
Colposcopy (≤24 years)						0.055
	Low-grade	22 (38.6)	19 (22.1)	6 (21.4)	9 (17.6)	
	High-grade/Cancer	35 (61.4)	67 (77.9)	22 (78.6)	42 (82.4)	
	Total	57	86	28	52	
Colposcopy (≥25 years)						0.533
	Low-grade	10 (21.7)	19 (22.6)	1 (6.7)	8 (18.2)	
	High-grade/Cancer	36 (78.3)	65 (77.4)	14 (93.3)	36 (81.8)	
	Total	46	84	15	44	
Histopathology (≤24 years)						0.190
	Negative/low-grade	19 (59.4)	35 (57.4)	10 (43.5)	16 (39)	
	High-grade/Cancer	13 (40.6)	26 (42.6)	13 (56.5)	25 (61)	
	Total	32	61	23	41	
Histopathology (≥25 years)						0.003
	Negative/low-grade	27 (58.7)	41 (48.8)	3 (20)	11 (25.6)	
	High-grade/Cancer	19 (41.3)	43 (51.2)	12 (80)	32 (74.4)	
	Total	46	84	15	43	

* χ^2^ test, p<0.05.

Evaluation of the associations between lesion severity and age suggested that low-grade lesions predominated at the extremes of age, whereas high-grade lesions were more prevalent in patients aged 21–35 years, especially in patients aged ≥25 years (Tables 3 and 4).

**Table 3:** Comparison of the four groups defined by age in relation to the severity of cytopathology, colposcopy, and histopathology

		<21 years	21–24 years	25–35 years	>35 years	p value*
Cytopathology						<0.001
	Low-grade	68 (68.7)	77 (55.0)	59 (40.7)	25 (56.8)	
	High-grade	31 (31.3)	63 (45.0)	86 (59.3)	19 (43.2)	
	Total	99	140	145	44	
Colposcopy						0.005
	Low-grade	30 (32.6)	26 (20)	23 (15.9)	15 (34.1)	
	High-grade/cancer	62 (67.4)	104 (80)	122 (84.1)	29 (65.9)	
	Total	92	130	145	44	
Histopathology						0.008
	Low-grade	34 (59.6)	46 (46)	55 (38.2)	27 (61.4)	
	High-grade	23 (40.4)	54 (54)	89 (61.8)	17 (38.6)	
	Total	55	100	120	32	

* X^2^ test, p<0.05.

**Table 4:** Comparison of the two groups defined by age in relation to the severity of cytopathology, colposcopy, and histopathology

		≤24 years	≥25 years	p value^*^
Cytopathology				0.001
	Low-grade	145 (60.7)	84 (44.4)	
	High-grade	94 (39.3)	105 (55.6)	
	Total	239	189	
Colposcopy				0.240
	Low-grade	56 (25.2)	38 (20.1)	
	High-grade/cancer	166 (74.8)	151 (79.9)	
	Total	222	189	
Histopathology				0.194
	Low-grade	80 (51)	82 (43.6)	
	High-grade	77 (49)	106 (56.4)	
	Total	157	188	

* Exact Fisher test, p<0.05.

OR=1.93 (95%CI=1.31 – 2.84)=odds in favor of having high-grade cytopathology in women aged ≥25 years compared to women aged ≤24 years.

## DISCUSSION

This study suggests that the colposcopic size of cervical lesions is directly related to lesion severity, regardless of age. However, only patients aged ≥25 years had a direct relationship between lesion size and histopathological severity. In patients ≤24 years, a correlation was found only with cytopathological features. Histopathology showed the strongest association between severity and lesion size. This is a positive aspect of this study because histopathology is the gold standard for diagnosing precursor lesions and cervical cancer. Notably, no other studies correlating the cited characteristics with age were found. Kierkegaard et al.^(23)^ analyzed the relationship between histopathological severity of the lesions and specific colposcopic findings (*e.g*., transformation zone size, lesion size, margins, vessels, and intensity of acetate whitening of the lesions) of 896 women between 15 and 71 years (mean age, 27.3 years) and concluded that lesion size was an independent prognostic factor for lesion progression and that it was related to the severity of the histopathological lesions. Jarmulowicz et al.^(11)^ analyzed the correlation between the severity of the cervical smear and lesion size in 70 women with changes in cytopathology and histopathology of a surgical specimen considered technically satisfactory. They showed that the size of the histopathological lesion may affect the cervical smear severity. The authors showed that mean dyskaryosis could be the cutoff point to explain the discrepancies between cytopathology, colposcopy, and histopathology. In contrast, Nam et al.^(14)^ found no association between cytopathological severity and colposcopic lesion size.

In this study, cytopathology for women ≤24 years showed that low-grade lesions occupied ≤3 quadrants of the cervix. Low-grade lesions in adult women tended to occupy ≤2 quadrants. Jarmulowicz et al.^(11)^ observed that low-grade cytopathology occupied up to 2 quadrants of the cervix, regardless of age. The colposcopic findings for the two age groups showed higher grade (high-grade or cancer) in lesions occupying ≥3 quadrants. Kierkegaard et al.^(23)^ reported that a lesion that spans over ≥2 quadrants of visibility in the cervix has an Odds Ratio to determine a high-grade lesion of 3.6. High-grade or cancer histopathology prevailed in lesions occupying ≥3 quadrants of the cervix for patients ≤24 years and for women ≥25 years. Bowring et al.^(22)^ reported an association between lesions occupying ≥2 quadrants and the histopathological diagnosis of cervical intraepithelial neoplasia 2 or more in 70% of the cases. Munmany et al.^(13)^ reported that lesions occupying only one quadrant of the cervix have a greater chance of presenting with negative histopathology.

This study has several strengths. We adopted a reference standard to identify the target conditions. Patients who required a histopathological sample were subjected to the same gold standard, and non-interpretable or intermediate results were reported. Moreover, the statistical tests were adjusted to minimize chance and bias, and patients with clinical conditions that could alter or impair the measurement of the lesion area below the surface of the cervix were excluded from the study, an issue that is unsettling and less discussed so far.

However, this study had some limitations. Retrospective studies are considered less reliable in reflecting clinical practice because examinations were not performed with the objective of evaluation. The target population was recruited from a tertiary hospital and was less representative of the reality (an issue that did not affect the objectives of the study). Finally, it was not possible to determine whether patients were subjected to the tests within a short period to ensure that the evaluated conditions remained unchanged.

## CONCLUSION

In conclusion, we found that lesion size was directly related to the severity of cytopathological, colposcopic, and histopathological lesions, regardless of age. However, only patients aged ≥25 years had a direct relationship between lesion size and histopathologic severity. Patients aged ≤24 years showed a correlation with cytopathology only.
